# Exploring the motivation of health professionals to engage with research at various career stages

**DOI:** 10.1186/s12913-024-10772-z

**Published:** 2024-03-07

**Authors:** Louisa M. D’Arrietta, Venkat N. Vangaveti, Melissa J. Crowe, Bunmi S. Malau-Aduli

**Affiliations:** 1 College of Medicine and Dentistry, James Cook University, Townsville, QLD 4811 Australia; 2 Library Services, Townsville University Hospital, Townsville Hospital and Health Service, Townsville, QLD Australia; 3 Townsville Institute of Health Research and Innovation, Townsville University Hospital, Townsville Hospital and Health Service, Townsville, QLD Australia; 4 College of Healthcare Sciences, James Cook University, Townsville, QLD Australia; 5 School of Medicine and Public Health, University of Newcastle, New South Wales, Australia

**Keywords:** Career stage, Motivation, Barriers, Facilitators, Expectancy-value-cost theory, Research

## Abstract

**Background:**

Research is crucial for improved healthcare and better patient outcomes, but there is a current shortage of clinician-researchers who can connect research and practice in the health professions field. This study aimed to investigate the effect of career stage, previous training and involvement in research on health professionals' (HPs) motivations to engage in research while in public hospital clinical roles. HPs' perceived motivation concerning the importance, value, and barriers attributed to research during different career stages were examined.

**Methods:**

A mixed methods study design was adopted for this research. An online survey developed based on the Expectancy-Value-Cost (EVC) theory was distributed to HPs (doctors, nurses, midwives, and allied health professionals) in three North Queensland Public Hospitals. Data analysis included descriptive and inferential statistics for the quantitative data and content analysis for the qualitative text responses.

**Results:**

Three hundred and fifty-five responses were received. Prior research training and involvement in research influenced respondents' perceptions about the importance, attitude, motivators, and barriers to research. Attainment value was the overarching motivation for involvement in research and research training for all career stages and all professional HP groups. Positive attitude to research was significantly higher (*P* = 0.003) for the allied health group (27.45 ± 4.05), followed by the medical (26.30 ± 4.12) and then the nursing and midwifery group (25.62 ± 4.21). Perceived importance and attitude attributed to research were significantly higher (*P* < 0.05) for those who had research training (26.66 ± 3.26 and 28.21 ± 3.73) compared to those who did not have research training (25.77 ± 3.77 and 23.97 ± 3.53). Significantly higher (*P* < 0.05) perceptions of organisational and individual barriers were reported among early career (50.52 ± 7.30) respondents compared to their mid-career (48.49 ± 8.14) and late career (47.71 ± 8.36) counterparts.

**Conclusion:**

The findings from this study provide valuable insights into the factors that influence HPs' motivation for research. The results underscore the importance of professional group, involvement in research, exposure to research training, career stage, gender, and organisational support in shaping HPs' attitudes, values, and perceived barriers to research. Understanding these factors can inform the development of targeted strategies to enhance research engagement among HPs and promote evidence-based practice in healthcare.

**Supplementary Information:**

The online version contains supplementary material available at 10.1186/s12913-024-10772-z.

## Background

Vocational psychology and sociology literature [1] have revealed the importance of career stage as a motivator for human behaviour in the work environment [1]. Career stage theories purport that over the career trajectory, individuals transition through different career stages, which reflect their needs, attitudes, values, and concerns [2, 3]. Individuals establishing and exploring their career may be categorised as early career; those seeking continuous improvement and stability as mid-career; and those with maintained or declining performance as late career [2].

In the contemporary era, career stage has been identified as fundamental to health professionals' engagement with research [4]. Hence, the Australian Government has recently outlined its intention to invest 200 million Australian dollars (AUD) over the next 10 years commencing 2022–23 to support the next generation of talented Australian researchers [4]. This initiative aimed to address forebodings from a 2016 national survey which showed that 83% of researchers considered leaving for another career, with the majority being mid-career researchers, a loss which would significantly impact Australia's research knowledge and skills base [4]. Therefore, research capacity building has focused on creating career trajectories or pathways to enhance participation in research and has embraced research training as the beginning of what has traditionally been called the training pipeline [5] to foster research readiness [6]. The goals are to support health professionals (HPs) researching topics important to clinical care [7], "increase the number of research-focused clinicians working to solve health problems and increase the number of professionals transitioning from early to mid and late-stage health and medical research careers" [4].

As HPs transition through distinct career stages, they have unique career concerns and personal and psychological needs, which may change as they move between career stages [1, 3]. These have been identified in the literature to include intrinsic motivation, work-life balance, inclusiveness, work environment, mentorship, and funding availability [8]. Enabling transitioning between research career stages is recognised as an effective strategy to build research capital [4, 5]. Unfortunately, there has been a continual decline in research-active clinicians, first mooted over 40 years ago [9], which continues to be of paramount concern [10]. While it may be desirable for all HPs to be engaged in research, only a minority of HPs are involved in research in Australia [11] as not all have an appetite or desire to do so [12].

Developing and sustaining a strong health research workforce, beginning with young and emerging researchers through to mid-career researchers by enhancing skills and capabilities, has become the focus of research capacity building [13]. The decline has mostly impacted the mid-career research stage and has, therefore, most recently attracted special attention [5, 13, 14]. According to the Association of Australian Medical Research Institutes, "If we do not invest in the future of our mid-career researchers, we will see those current and future stars leaving research or moving to take up better supported overseas opportunities. These people are Australia's most valuable assets and must be protected and prioritised" [14]. A new approach is needed to aid identification of effective strategies for improving retention of HPs in research and supporting clinical research career trajectories and the embedded research workforce [15].

Medical, nursing and midwifery, and allied health professionals undertaking research have traditionally faced many challenges during their career stages emanating from the personal, professional, cultural, and organisational environments in which they work [2, 3, 16–19]. Understanding perceived motivations and barriers to engage in research at the micro level may facilitate alleviation of the problem at the macro level. One of the overriding barriers to HPs undertaking research has been recently articulated. "Clinical professionals usually have to choose between life as a researcher or a clinician” [4]. “This creates a divide between the kind of research happening in a lab and the research needed to improve clinical practice" [4]. Another barrier is the influence of early-career gender differences that have long been outlined in the literature especially for the medical profession in terms of unequal financial rewards [20] and time spent on parenting and domestic responsibilities [21] as obstacles to engaging with research training and research. Other challenges that particularly confront early career HPs are finding ways to balance work/life demands, mentors who can provide support and guidance and the increasingly time-consuming and demanding requirements to maintain clinical credentials [22]. Challenges for those in remote hospital settings are compounded by their isolation and include limited face-to-face professional identity and educational opportunities [23, 24], including professional isolation, large professional load, insufficient supervision [23], and limited opportunities to build skills and access career pathways [25].

The projected loss reflects the many barriers to research encountered at all career stages and would suggest that urgent further investigation is required, particularly about the values held about research at the individual and organisational level, juxtaposed by the facilitators that enable opportunities for research to ameliorate this dire foreboding. HPs' participation in research is largely underpinned by their perception of the importance and personal values they attribute to research [12]. How these values are engendered depends on the systematic evolution of their career pipeline encouraged by research training and the organisational research culture in which HPs feel embedded on an individual and group basis [15]. Therefore, at the top of the research agenda is the need to understand how career stage affects motivation [16]. The Expectancy-Value-Cost (EVC) theory [26, 27] offers a unique perspective and new lens with which to examine the space of research for HPs and may provide some clarity regarding considerations of the motivations and barriers to engaging with research at different career stages. Studies have shown that value attributed to research is the key factor regarding motivation to undertake research [6, 28] Value is interpreted as either attainment value (i.e., the importance of doing well), intrinsic value (i.e., personal enjoyment) and utility value (i.e., perceived usefulness for future goals); and the value HPs place on the outcome largely drives their motivation to achieve it [29, 30].We contextualised the Expectancy-Value-Cost Model of Motivation (EVC) [26, 27] to investigate the expectations, values and costs attributed to HPs engaging in research and its role in Motivation (M) for uptake and continuation of research by HPs in early, mid and late career stages and its application in research capacity building. Through the EVC theory lens, this study therefore sought to answer three research questions:

1. What factors influence motivation for research?
2. What is the impact of research training and level of involvement on value attributed to research at different career stages?
3. What is the effect of career stage on the individual and organisational barriers that impede motivation for research?

## Methods

### Study design

This study employed a mixed methods design [31], combining quantitative and qualitative data to explore the perceptions of HPs from three public hospitals in North Queensland regarding factors influencing motivation for research, the impact of research training at different career stages, and the effect of career stage and gender on individual and organisational barriers to research motivation.

The use of a mixed methods design was deemed appropriate for this study as it allows for a comprehensive exploration of the research questions by combining the strengths of both quantitative and qualitative approaches [31]. The quantitative component (cross-sectional online survey) provided numerical data to identify patterns and relationships, while the qualitative component (content analysis of open-ended responses) offered a deeper understanding of the participants' perceptions and experiences. The Expectancy-Value-Cost (EVC) theory served as a guiding framework, providing a structured foundation for assessing motivation factors. Cross-sectional studies give a profile of the sample population at one point in time and allow examination of associations between variables; while in-depth exploration of open-ended responses from online surveys using content analysis techniques aid derivation of meaningful insights from textual data [32].

The quantitative phase of the study (Expectancy-Value-Cost theory based online survey) served as the primary data collection method, prioritised for its ability to generate numerical data efficiently. The survey allowed for a large-scale assessment of HPs’ perceptions. The qualitative component, involving content analysis of open-ended survey responses, was conducted sequentially. This allowed for an in-depth exploration of the factors identified in the quantitative phase. Integration occurred during the interpretation phase in the Discussion, where quantitative and qualitative findings have been synthesised to provide a comprehensive understanding of the research questions. This study has been reported according to the Good Reporting of A Mixed Methods Study (GRAMMS) criteria for reporting mixed methods research [33] (See Supplementary Material 1).

Ethics approval for the study was obtained from The Human Research Ethics Committee (HREC) of Townsville Hospital and Health Service (Reference number: HREC/2019/QTHS/59607) and James Cook University Human Ethics Committee, Australia (H8314). All participants were provided with an information sheet as the first page of the online survey that detailed the aims of the study and the ethical obligations of the researchers which included confidentiality, informed consent, and publication of anonymised responses. All these obligations were strictly adhered to during the research process. Informed consent was implied by submission of the completed online survey.

### Data collection

Prospective participants were a North Queensland population of early career, mid-career, and late career allied health, medical, nursing and midwifery professionals working in public regional, rural-remote hospitals in Cairns Hospital and Hinterland Health Service (CHHHS), Mackay Hospital and Health Service (MHHS), and Townsville Hospital and Health Service (THHS). A total population of 10,704 HPs, including 1,775 allied health practitioners, 1,833 medical and 7,096 nurses and midwives were invited to participate. An a priori sample size calculation conducted using Open Epi (Version 3), indicated that 254 participants were required to achieve 90% power for detecting a medium-sized effect with 0.05 level of statistical significance. The first named author (LMDA) sent the survey through the site co-ordinators at the three sites to participants. Anonymous survey responses were collected via online Survey Monkey® (SVMK Inc.) from November 2020 to March 2021. To increase the response rate [34, 35], reminders were sent to prospective participants thrice during the data collection period. Additionally, an incentive of an e-card to the value of $25.00 or their donation to the Children's Brain Cancer Centre, Children's Hospital Foundation Brisbane was offered to respondents for participating in the study in appreciation of the estimated 15 min required to complete the survey. The uptake of the offer was 165 (46.5%) donation, 150 (42.3%) e-card and 40 (11.3%) not nominated.

### Survey instrument development

The survey instrument (Supplementary Material 2) was adapted from previously validated questionnaires [36–40] and developed based on the EVC model with a focus on the factors identified by the authors in a recent systematic review [12] within the three domains: Expectancy for research capacity, Value reflected in attitude, and Cost which relates to barriers. The survey comprised three (3) major parts. Part A focused on the demographics of the participants. Part B had five sections (total of 57 questions) and utilised 4/5-point Likert scale questions (from 1 = strongly disagree to 4/5 = strongly agree) to assess participants' perceptions of the motivators and de-motivators to undertaking research. Part C included open-ended questions relating to enablers and barriers to undertaking/continuing with research. These scores were aggregated for data analysis. To ensure shared understanding of the purpose of the study, a definition of research experience/training was provided in the survey. This was defined as ‘the required skills, knowledge and information available pertaining to successfully conducting research’. The survey was pilot tested for content validity by a representative group of 20 HPs of similar backgrounds to those whom the survey was distributed. Inter-item correlations and internal consistency indexes confirmed the reliability of the instruments' items and scales. The survey instrument had an acceptable reliability with an overall Cronbach's alpha score of 0.861.

### Data analysis

Participants' quantitative responses were analysed using SPSS software version 28 (IBM Corp. Released 2021. IBM SPSS Statistics for Windows, Version 28.0. Armonk, NY: IBM Corp). Numerical data were presented as frequencies, means and standard deviations/standard errors, while categorical data were presented as frequencies and proportions. Likert scale items were treated as ordinal data. However, to assess the influence of the variables on motivation for research, a total score which is the sum of the item scores was calculated for perceived importance of and attitude to research. While these total scores are discrete and not continuous, under the Likert perspective, these total scores were treated as ordinal approximation of a continuous variable [41, 42]. Upon normality check, appropriate parametric (Student's t-test or one way ANOVA /two-way ANOVA) and non-parametric tests (Mann Whitney U/ Kruskal Wallis) were conducted followed by Bonferroni *post-hoc* test for multiple testing. *P* value of ≤ 0.05 was considered statistically significant. Content analysis [32] was utilised to identify frequency of occurrence of concepts from the responses to the open-ended questions noting the three (3) most mentioned for each question. This process was completed independently by LMDA and BMA and it involved four iterative steps—decontextualisation, recontextualisation, categorisation and compilation [43, 44]. In the decontextualisation stage, the researchers (LMDA and BMA) familiarised themselves with the data (read through the transcribed text) to understand the data and assigned codes. The recontextualisation stage involved both LMDA and BMA checking together to ensure that all aspects of the content had been addressed with regard to the aims of the study. The original text was reread with the final list of codes, and any missed relevant text was included. The codes (meaning units) were condensed in the categorisation stage, and themes and categories were identified. The themes were established in the final—compilation stage [44]. Validation of the themes was sought from the other researchers to ensure rigour and validity. Illustrative quotes are presented and affixed with participants' survey number and demographic profiles. For example, P151 FNMMC refers to Participant 151, Female, Nurse-Midwife, Mid-career.

## Results

### Characteristics and research experience of participants

A total of 355 participants completed the survey. Table 1 portrays the demographic characteristics of the participants. Respondents were predominantly females (78.6%), over half held postgraduate qualifications (60.6%), the majority were in their early (36.9%) to mid-stages (38.0%) of their careers. About half (53.0%) of the participants were within the 20–40 years age range. Nursing and midwifery professionals made up half (50.7%) of the respondents, followed by the allied health (29.0%), and the medical (20.3%) groups. Half of the respondents had not worked in major city hospitals, while just under half indicated that the opportunity to engage in research had or would influence their choice of hospital/workplace. About half (49.3%) of the respondents acknowledged that research activities were included in their role description. Over half of the respondents reported their involvement in research (57.3%) and research training (53.0%). Of those who had research training, the majority had found it a positive experience, but reported that research training was not obtainable in their current hospital system/ workplace. Overall, most respondents (75.4%) indicated that regardless of whether they had research training or not they would be interested in undertaking research training in the future.

**Table 1:** Characteristics and research experience of participants (n = 355)

**Variable**	**Total Nos (%) [Male**♂**: Female**♀** %]**
Gender	
• Male	76 (21.4)
• Female	279 (78.6)
Age	
• 20–30 Years	89 (25.0) [♂22.5; ♀77.5]
• 31–40 Years	98 (28.0) [♂25.5; ♀74.5]
• 41–50 Years	82 (23.0) [♂15.9; ♀84.1]
• 51-and above	86 (24.0) [♂20.9; ♀79.1]
Profession	
• Allied Health	103 (29.0) [♂25.0; ♀75.0]
• Medical	72 (20.3) [♂50.0; ♀50.0]
• Nursing/Midwifery	180 (50.7) [♂8.0; ♀92.0]
Completion of qualification	
• Australia	295 (83.1) [♂20.0; ♀80.0]
• Overseas	60 (16.9) [♂30.0; ♀70.0]
Qualification	
• Postgraduate	60.6 [♂27.0; ♀73.0]
• Undergraduate	39.4 [♂12.0; ♀88.0]
Career stage	
• Early career	37.3 [♂25.0; ♀75.0]
• Mid-career	38.5 [♂19.0; ♀81.0]
• Late career	24.2 [♂19.0; ♀81.0]
Work location	
• CHHHS	2.8 [♂20.0; ♀80.0]
• MHHS	16.9 [♂13.0; ♀87.0]
• THHS	80.3 [♂23.0; ♀77.0]
Have you previously worked in a hospital located in a major city?	
• Yes	49.6 [♂26.0; ♀74.0]
• No	50.4 [♂17.0; ♀83.0]
Has/ would the opportunity to engage in research influence your choice of hospital/workplace in which to practice?	
• Yes	42.0 [♂32.0; ♀68.0]
• No	58.0 [♂14.0; ♀86.0]
Are research related activities included in your role description?	
• Yes	49.3 [♂22.0; ♀78.0]
• No	50.7 [♂20.0; ♀80.0]
Are you/have you been involved in undertaking research**?**	
• Yes	57.3 [♂29.0; ♀71.0]
• No	42.7 [♂10.0; ♀90.0]
Have you had research training? (Defined as the required skills, knowledge, and information available pertaining to successfully conducting research)	
• Yes	53.0 [♂27.0; ♀73.0]
• No	47.0 [♂16.0; ♀84.0]
If yes, would you describe this as a positive experience?	
• Yes	84.2 [♂28.0; ♀72.0]
• No	15.8 [♂24.0; ♀76.0]
If yes, has the research training motivated you to undertake research?	
• Yes	63.4 [♂28.0; ♀72.0]
• No	36.6 [♂27.0; ♀73.0]
Was the research training in the hospital system for which you currently work?	
• Yes	26.2 [♂23.0; ♀77.0]
• No	73.8 [♂29.0; ♀71.0]
Would you be interested in undertaking research training in the future?	
• Yes	75.4 [♂20.0; ♀80.0]
• No	24.6 [♂20.0; ♀80.0]

### Factors that influence HPs' motivation for research

Table 2 portrays how the participants' professional group, career stage, previous exposure to research training and involvement in research influenced their responses about importance, attitude, and barriers to research. The most impacted was attitude to research, which was influenced by professional group, involvement in research and exposure to research training. Positive attitude to research was significantly higher (*P* = 0.003) for the allied health group (27.45 ± 4.05) compared to the medical group (26.30 ± 4.12) which was significantly higher than the nursing and midwifery group (25.62 ± 4.21). Attitude to research was also influenced by involvement in research and exposure to research training. Respondents who had undertaken research reported significantly higher (*P* = 0.001) positive attitude (27.63 ± 4.10) compared to those who had not (24.42 ± 3.63). Similarly, respondents who had research training reported significantly higher (*P* = 0.001) positive attitude (28.21 ± 3.73) compared to those who didn't have research training (23.97 ± 3.53).

**Table 2:** Factors that influence HPs' motivation for research

**Variable**	**Importance of research**	**Attitude to research**	**Motivators for research**	**Barriers**
**Professional Group**	*P* = 0.166	*P* = 0.003	*P* = 0.040	*P* = 0.083
Allied Health	25.98 ± 3.69	27.45 ± 4.05^a^	39.03 ± 4.73^a^	48.02 ± 7.48
Medical	25.77 ± 3.44	26.30 ± 4.12^ab^	37.08 ± 5.61^b^	48.28 ± 8.39
Nursing and Midwifery	26.61 ± 3.44	25.62 ± 4.21^b^	38.81 ± 5.30^ab^	50.07 ± 7.92
**Career stage**	*P* = 0.824	*P* = 0.280	*P* = 0.828	*P* = 0.029
Early career	26.38 ± 3.61	25.87 ± 3.77	38.72 ± 5.31	50.52 ± 7.30^a^
Mid-career	26.11 ± 3.20	26.52 ± 4.35	38.63 ± 4.70	48.49 ± 8.14^ab^
Late career	26.31 ± 3.85	26.74 ± 4.46	38.27 ± 5.63	47.71 ± 8.36^b^
**Gender**	*P* = 0.107	*P* = 0.001	*P* = 0.047	*P* = 0.001
Male	26.86 ± 3.43	28.05 ± 3.72	38.91 ± 5.43	46.23 ± 7.92
Female	26.10 ± 3.54	25.83 ± 4.21	38.40 ± 5.20	49.85 ± 7.78
**Are you/have you been involved in undertaking research?**	*P* = 0.122	*P* = 0.001	*P* = 0.234	*P* = 0.001
Yes	26.51 ± 3.57	27.63 ± 4.10	38.80 ± 5.16	47.63 ± 7.96
No	25.90 ± 3.44	24.42 ± 3.63	38.08 ± 5.37	51.21 ± 7.43
**Have you had research training**	*P* = 0.020	*P* = 0.001	P = 0.112	*P* = 0.032
Yes	26.66 ± 3.26	28.21 ± 3.73	38.92 ± 5.20	48.25 ± 8.35
No	25.77 ± 3.77	23.97 ± 3.53	37.98 ± 5.29	50.14 ± 7.29

Data are presented as mean ± SD. *P* values are reported for group analysis. Student's t- test was conducted for previous research involvement and research training. For career groups and professional groups, one way ANOVA was conducted followed by post-hoc test with Bonferroni correction. Within multi-group variables, values with different superscripts (^a, b, ab^) are significantly different

Perceived importance attributed to research was significantly higher (*P* = 0.020) for those who had research training (26.66 ± 3.26) compared to those who didn't have research training (25.77 ± 3.77). Motivators for research were significantly higher (*P* = 0.04) for the allied health professionals (39.03 ± 4.73), compared to the medical group (37.08 ± 5.61). Perceived barriers to engaging in research were significantly influenced by career stage, involvement in research and exposure to research training. Early career respondents reported significantly higher (*P* = 0.029) perceptions of barriers (50.52 ± 7.30) compared to their mid-career (48.49 ± 8.14) and late career (47.71 ± 8.36) counterparts. Additionally, respondents who had not undertaken research reported significantly higher (*P* = 0.001) barriers (51.21 ± 7.43) compared to those who had been involved in research (47.63 ± 7.96). Similarly, those who did not have previous research training reported significantly (*P* = 0.032) more barriers (50.14 ± 7.29) compared to those who had (48.25 ± 8.35).

### Influence of involvement in research on value attributed to research

Table 3 shows that value attributed to research was influenced by level of involvement in research, career stage, gender and type of professional group. Early, mid and late career participants who were involved in research reported significantly higher (*P* < 0.05) attainment value for research compared to their counterparts who were not involved in research. Additionally, early career participants who were involved in research reported significantly higher (*P* < 0.001) intrinsic value for research (52.43 ± 0.88) compared to their counterparts (48.21 ± 0.85) who were not involved in research. Furthermore, among those who were involved in research, early career respondents reported significantly higher (*P* < 0.001) attainment value (53.82 ± 0.77) for research compared to their late career counterparts (50.93 ± 0.77).

**Table 3:** Influence of involvement in research on the type of value (attainment, intrinsic and utility) attributed to research by different demographic groups (career stage, gender, and professional groups)

	Attainment		*P* value	Intrinsic		*P* value	Utility		*P* Value
Characteristic	No research involvement	Had research involvement		No research involvement	Had research involvement		No research involvement	Had research involvement	
**Career stage**									
**Early**	49.19 ± 0.74	53.82 ± 0.77	** < 0.001**	48.21 ± 0.85	52.43 ± 0.88	** < 0.001**	41.40 ± 0.53	40.91 ± 0.55	0.528
**Mid**	49.46 ± 0.85	51.69 ± 0.69	**0.045**	50.48 ± 0.98	51.97 ± 0.79	0.242	41.55 ± 0.61	40.09 ± 0.50	0.069
**Late**	46.42 ± 1.34	50.93 ± 0.77	**0.004**	49.68 ± 1.54	51.25 ± 0.88	0.377	41.00 ± 0.97	40.58 ± 0.55	0.712
**Gender**									
**Male**	49.76 ± 1.67	53.41 ± 0.81	**0.051**	51.53 ± 1.88	53.52 ± 0.91	0.343	40.23 ± 1.17	39.76 ± 0.57	0.720
**Female**	48.70 ± 0.56	51.38 ± 0.51	** < 0.001**	48.98 ± 0.63	51.13 ± 0.58	**0.013**	41.56 ± 0.39	40.82 ± 0.36	0.169
**Professional group**									
**AH**	48.34 ± 1.19	51.67 ± 0.74	**0.019**	50.00 ± 1.34	52.67 ± 0.83	0.092	41.96 ± 0.81	41.04 ± 0.51	0.342
**M**	47.09 ± 1.83	51.89 ± 0.80	**0.017**	46.72 ± 2.06	50.57 ± 0.90	0.088	39.36 ± 1.25	39.01 ± 0.55	0.801
**N&M**	49.15 ± 0.63	52.34 ± 0.74	**0.001**	49.32 ± 0.71	52.04 ± 0.84	**0.014**	41.52 ± 0.43	41.27 ± 0.51	0.712

Data are presented as mean ± SE. *P* values are reported for group analysis from post hoc analysis. Student's t- test was conducted for previous research involvement. For career stage and professional groups, two-way ANOVA was conducted followed by post-hoc test with Bonferroni correction.

*AH*  allied health, *M*  medical, *N&M*  nursing and midwifery

Male and female participants who were involved in research also reported significantly higher (*P* ≤ 0.05) attainment value for research compared to their counterparts who were not involved in research. Additionally, female participants who were involved in research reported significantly higher (*P* = 0.013) intrinsic value for research (51.13 ± 0.58) compared to their counterparts (48.98 ± 0.63) who were not involved in research. Male participants who were involved in research reported significantly higher (*P* < 0.05) attainment value (53.41 ± 0.81) vs (51.38 ± 0.51) and intrinsic value (53.52 ± 0.91) vs (51.13 ± 0.58) compared to their female counterparts.

Similarly, participants from all professional groups who were involved in research had significantly higher (*P* < 0.01) attainment value than their non-research active counterparts. Intrinsic value was also significantly higher (*P* = 0.014) for the nursing and midwifery group (52.04 ± 0.84) who were involved in research in comparison to their counterparts who were not involved in research.

Overall, Table 3 shows that involvement in research significantly increased the participants' perceptions of the attainment value, and to a lesser extent, the intrinsic value of research. Intrinsic value was highly significant for the early career, female and nursing and midwifery respondents who had research involvement, compared to their respective counterparts.

### Influence of research training on motivation for research

Table 4 presents the influence of research training on value attributed to research for different demographic groups (career stage, gender, and professional group). Early career participants who had been exposed to research training reported significantly higher (*P* < 0.05) attainment (53.26 ± 0.75 vs 49.60 ± 0.75) and intrinsic (51.51 ± 0.85 vs 48.98 ± 0.85) values compared to their respective counterparts who did not have research training. Late career participants who had been exposed to research training reported significantly higher (*P* < 0.05) attainment (52.06 ± 0.86 vs 46.48 ± 1.05), intrinsic (53.28 ± 0.98 vs 47.29 ± 1.19) and utility (41.65 ± 0.61 vs 39.25 ± 0.74) values compared to their respective counterparts who did not have research training.

**Table 4:** Influence of research training on the type of value (attainment, intrinsic and utility) attributed to research by different demographic groups (career stage, gender, and professional groups)

	Attainment		*P* value	Intrinsic		*P* value	Utility		*P* Value
Characteristic	No research training	Had research training		No research training	Had research training		No research training	Had research training	
**Career stage**									
**Early**	49.60 ± 0.75	53.26 ± 0.75	** < 0.001**	48.98 ± 0.85	51.51 ± 0.85	**0.038**	40.81 ± 0.53	41.51 ± 0.53	0.356
**Mid**	49.68 ± 0.84	51.57 ± 0.70	0.088	50.16 ± 0.96	52.21 ± 0.79	0.101	42.18 ± 0.59	39.64 ± 0.49	**0.001**
**Late**	46.48 ± 1.05	52.06 ± 0.86	** < 0.001**	47.29 ± 1.19	53.28 ± 0.98	** < 0.001**	39.25 ± 0.74	41.65 ± 0.61	**0.014**
**Gender**									
**Male**	49.84 ± 1.37	53.83 ± 0.85	**0.029**	52.21 ± 1.53	53.51 ± 0.95	0.142	39.05 ± 0.97	40.16 ± 0.60	0.332
**Female**	48.57 ± 0.54	51.63 ± 0.52	0.390	48.32 ± 0.60	51.86 ± 0.58	**0.019**	41.21 ± 0.38	41.11 ± 0.37	0.856
**Professional group**									
**AH**	48.59 ± 1.16	51.62 ± 0.74	**0.029**	49.81 ± 1.29	52.78 ± 0.82	0.054	41.81 ± 0.80	41.09 ± 0.51	0.448
**M**	49.04 ± 1.25	52.17 ± 0.89	**0.043**	49.13 ± 1.40	50.37 ± 1.00	0.469	38.30 ± 0.86	39.46 ± 0.62	0.277
**N&M**	48.71 ± 0.63	52.89 ± 0.73	** < 0.001**	48.48 ± 0.70	53.14 ± 0.82	** < 0.001**	41.31 ± 0.43	41.55 ± 0.50	0.728

Data are presented as mean ± SE. *P* values are reported for group analysis from post hoc analysis. Student's t- test was conducted for previous research training. For career stage and professional groups, two-way ANOVA was conducted followed by *post-hoc* test with Bonferroni correction values as shown

*AH*  allied health, *M*  medical, *N&M*  nursing and midwifery

Male participants who had been exposed to research training reported significantly higher (*P* < 0.029) attainment value (53.83 ± 0.85 vs 49.84 ± 1.37) compared to their no research training counterparts. Similarly, female participants who had been exposed to research training reported significantly higher (*P* < 0.019) intrinsic value (51.86 ± 0.58 vs 48.32 ± 0.60) compared to their no research training counterparts. Table 4 also shows that male participants reported significantly higher (*P* < 0.05) attainment and intrinsic values compared to their female counterparts.

Significantly higher (*P* < 0.05) attainment value was reported by all allied health, medical and nursing/midwifery professionals who had been exposed to research training compared to their respective no research training counterparts. Significantly higher (*P* < 0.001) intrinsic value was also reported for the nursing/midwifery group who had research training (53.14 ± 0.82 vs 48.48 ± 0.70) compared to their no research training counterparts.

Overall, research training significantly influenced the attainment value for the early and late career cohorts, males, and all the professional groups. Intrinsic value was significantly higher for early and late career cohorts, males and allied health and nursing and midwifery groups who were exposed to research training. Utility value was significant for mid-career, late career who had research training.

### Perceived barriers to research

The findings from the analysis of the HPs’ responses to the close- and open-ended survey questions were integrated and synthesised to provide a comprehensive understanding to answer research question 3. Table 5 reports the influence of career stage on participants' perceptions about barriers to research. Content analysis of the open-ended questions confirmed and aligned with the eight (8) barriers identified in Table 5. Five (5) out of the eight questions on organisational barriers to research were significantly influenced by career stage. Overall, early career HPs were more impacted by organisational barriers than their mid, and late career counterparts and lamented thus: *"It [research] needs to be given equal importance as service delivery". (P107 MMEC).*

**Table 5:** Influence of career stage on barriers to research

Barrier statements	*P* value	Early	Mid	Late
**Organisational barriers**				
Research is not valued in my work organisation	**0.017**	2.34 ± 1.11^b^	2.35 ± 0.99^b^	2.75 ± 1.18^a^
Lack of opportunity to research in my area of interest	**0.902**	3.01 ± 1.11	3.00 ± 1.03	3.07 ± 1.04
Lack of funding for research	**0.749**	3.51 ± 0.96	3.55 ± 0.90	3.61 ± 0.90
Lack of protected research time	**0.893**	4.04 ± 0.88	4.05 ± 0.83	4.09 ± 0.83
Lack of access to research space/ equipment	**0.031**	3.51 ± 1.03^a^	3.15 ± 1.10^b^	3.35 ± 1.01^ab^
Lack of research skills/ training/ support	**0.002**	3.75 ± 0.92^a^	3.41 ± 1.10^ab^	3.22 ± 1.24^b^
Lack of access to individuals with appropriate ethics, research governance and grants expertise	**0.037**	3.44 ± 1.02^a^	3.13 ± 1.17^b^	3.08 ± 1.16^b^
Lack of access to library services for assistance with literature searching and document supply	**0.050**	2.73 ± 1.01^a^	2.51 ± 1.00^ab^	2.39 ± 1.06^b^
**Individual barriers**				
I don't believe research would benefit patient care	**0.747**	1.44 ± 0.64	1.38 ± 0.56	1.41 ± 0.73
I don't believe research would benefit the organisation for which I work	**0.509**	1.49 ± 0.70	1.40 ± 0.55	1.40 ± 0.66
Difficulty in finding a research supervisor/mentor	**0.003**	3.41 ± 1.11^a^	2.96 ± 1.11^b^	3.04 ± 0.93^ab^
Achieving a work life balance	**0.044**	3.90 ± 0.91^ab^	3.95 ± 0.92^a^	3.61 ± 1.11^b^
Family & carer commitments	**0.193**	3.57 ± 1.04	3.68 ± 1.02	3.40 ± 1.15
Lower salary than a clinical career	**0.195**	3.40 ± 0.98	3.24 ± 0.89	3.17 ± 0.89
Job insecurity relative to a clinical career	**0.204**	3.56 ± 0.95	3.45 ± 0.92	3.32 ± 1.01
The thought of research makes me nervous	**0.001**	3.36 ± 1.16^a^	3.21 ± 1.30^ab^	2.71 ± 1.29^b^

Data are presented as mean ± SD. *P* values are reported for group analysis. Kruskal Wallis Test was conducted followed by post-hoc test with Bonferroni correction. Within each variable, values with different superscripts (^a, b, ab^) are significantly different

The most significant organisational barrier to research as reported by the early career group was 'lack of research skills/ training support'. Their scores were significantly higher for this barrier than the mid and late career HPs (*P* = 0.002) 3.75 ± 0.92 vs mid (3.41 ± 1.10) vs late career (3.22 ± 1.24).

*"I have research training from university, however none since working as a clinician. Barriers to attending training are line manager approval and busy caseloads. I would also be unsure what to use the information for without support/funding from the hospital and line manager."* (P 277 FAHEC).

However, the late career HPs reported stronger (*P* = 0.017) perceptions of research not being valued in their work organisation (2.75 ± 1.18) compared to the mid (2.35 ± 0.99) and early career (2.34 ± 1.11) HPs. These late career respondents offered some insights for improving organisational support to demonstrate the value held for research in terms of providing allocated time and career pathways for research.

*"I think the HHS needs to really value research. We say that we do, however, in reality, I do not believe this is the case. We have a growing number of research experienced health professionals on staff (e.g., with PhDs) but there's very few paid research positions for them. We are underutilising these people and their research skills*." (P92 FNMLC).

*"Practical encouragement in the form of enthusiasm from management, and time allocation for research could make a huge difference in my workplace". (P136 FNMLC).*

Only three (3) out of the eight questions on individual barriers to research were significantly influenced by career stage (Table 5). Early career HPs were also more impacted by individual barriers than their mid- and late career counterparts, except for 'difficulty in achieving work-life balance' where the mid-career HPs expressed significantly stronger (*P* = 0.044) perceptions (3.95 ± 0.92) compared to their early career (3.90 ± 0.91) and late career (3.61 ± 1.11) counterparts.

*"Work/life balance - such a tricky balance, I love clinical therapeutic work so this is my priority [but I] lack confidence in research methods. I would be interested in joining a team as an assistant and learning and then building up to lead research however there are limited opportunities to do this while maintaining a work life balance and [with] the same income."* (P 315 FAHMC).

The three topmost reasons given for non-participation in research training were (1) time costraints—30.0% (2) never given the opportunity—27.0% and (3) never knew it was available—19.0%. The three topmost discouragements from undertaking/continuing with research were (1) time constraints—62.0%, (2) not feeling supported by the organisation -18.6%, and lack of opportunity to engage in research—9.5%. The topmost recommendations from the respondents as the best motivators for undertaking/continuing with research were (1) increasing emphasis on importance of research in improving clinical care/patient care/treatment, (2) organisations providing protected research time and (3) having research valued in the workplace.

## Discussion

In this study, HPs' research motivation was investigated in terms of the perceived influence of involvement in research and research training on the values (attainment, intrinsic and utility) attributed to research at different career stages and by different gender and professional groups. This study builds on previous work [39] and its uniqueness is that it utilised the EVC model to investigate the effect of career stage on HPs motivation for, and barriers to, undertaking research. Gaining insight into what motivates HPs to undertake research training and research at different career stages is fundamental to building research capacity to encourage participation [45].

HPs′ exposure to research training lays the foundation for and underpins their confidence to undertaking research [6]. This study demonstrated that having had research training contributed significantly to participants' attitude and perceptions of importance and barriers to research. Furthermore, respondents who had previous research experience and research training reported more positive attitudes towards research compared to those without such experiences. This highlights the importance of hands-on research involvement and training in fostering a positive attitude towards research among HPs. In addition to attitude, the perceived importance attributed to research was also influenced by research training. Participants who had research training perceived research to be of higher importance compared to those without research training. This finding indicates that research training can enhance HPs' understanding of the significance and relevance of research in their professional practice.

Allied health professionals exhibited the most positive attitude towards research, followed by medical professionals and then nursing and midwifery professionals. This finding suggests that the nature of one's profession may play a role in shaping their attitude towards research. The study further reveals that motivators for research varied among professional groups. Allied health professionals showed higher motivation for research compared to medical professionals. This finding suggests that different professional groups may have distinct drivers and incentives to engage in research activities. Understanding these motivators can inform strategies to enhance research engagement among HPs [12, 46]. All professional groups, including allied health, medical, and nursing/midwifery professionals, showed significantly higher attainment value among those with research training compared to their counterparts without research training. The nursing and midwifery group, in particular, exhibited significantly higher intrinsic value among those with research training. These findings highlight the importance of research training in fostering a greater appreciation for research among different professional groups.

Additionally, research training significantly influenced the attainment value for early and late career participants. Early career participants and late career participants who had research training demonstrated higher attainment, intrinsic, and utility values compared to their counterparts without research training. This indicates that research training can contribute to a greater appreciation of research across different stages of a healthcare professional's career. Gender differences were also observed in the influence of research training on value. Male participants with research training exhibited higher attainment value compared to their counterparts without research training. Female participants with research training demonstrated higher intrinsic value compared to their counterparts without research training. Male participants also demonstrated higher attainment and intrinsic values than their female counterparts. These findings suggest that research training may have a differential impact on the perceived value of research based on gender. On this evidence, given that three quarters of the hospital's workforce is female, it behoves health organisations to improve research capacity among female HPs, in an effort for overall improvement of organisational research capacity and output [45].

Overall, involvement in research significantly increased the participants' perceptions of attainment value and intrinsic value of research. Early career participants who were involved in research demonstrated higher intrinsic value compared to their counterparts who were not involved in research. This finding suggests that active engagement in research can foster a sense of personal fulfillment and satisfaction among early career HPs. Gender differences were also observed in the value attributed to research. Male and female participants who were involved in research reported higher attainment value compared to their non-research active counterparts. Female participants involved in research displayed higher intrinsic value compared to their non-research active counterparts. This indicates that gender may interact with research involvement to influence the perceived value of research among HPs. Similarly, professional group influences the value attributed to research. Participants from all professional groups who were involved in research demonstrated higher attainment value compared to their non-research active counterparts. The nursing and midwifery group, specifically, showed higher intrinsic value among those involved in research compared to their non-research active and other professional counterparts as indicated in previous studies [12, 46]. These findings suggest that research involvement can enhance the perceived value of research across professional groups, with varying effects on different aspects of value.

Finally, this study examined the perceived barriers to research, with a focus on the influence of career stage. Triangulation of the quantitative and qualitative data in this study showed that barriers at various career stages can be influenced by the value espoused by HPs and re-enforced by the educational and organisational opportunities experienced as HPs navigate through the constructs of the organisational research culture [47, 48]. This study identified eight (8) specific barriers (five organisational and three individual) that are of significant concern to HPs at various career stages. Early career HPs reported greater impact from organisational and individual barriers compared to their mid-career and late career counterparts. Organisational barriers, such as a lack of research skills/training support, were particularly significant for early career HPs [49, 50]. This suggests that early career HPs may require targeted support and resources to overcome these barriers and engage in research [4, 5, 14, 51, 52]. On the other hand, mid and late career HPs expressed stronger perceptions of research not being valued in their work organisation. Major barriers identified were lack of protected research time, funding and opportunity to research in the area of interest. This highlights the need for organisational recognition and support for research at all career stages. Previous studies, social worth emphasises the prosocial aspect, which is particularly important at later career stages [2] and might therefore suggest suitability for including late career HPs in a mentorship role [53, 54].

Future studies could utilise qualitative research methods to explore how male and female HPs navigate their way through barriers at different career stages – early, mid-career, late career. Future explorations could also consider whether the three HP groups (allied health, medical, nursing and midwifery) follow similar or dissimilar trajectories in terms of how their research values change over their career stages, and whether having research experience or training is recognised by their organisations and if there are any advantages of having research training/degrees. Furthermore, investigating management’s understanding of the broader significance and worth (value) as well as the practical advantages and positive outcomes (benefits) of research and research training is warranted, especially in light of the participants' reported emphasis on involvement in research to improve clinical care and their percieved under-value of research within the organisation. While this concept falls beyond the current study's scope, it emerges as a crucial area for exploration in future research endeavours.

### Implications for practice

The findings of this study have several implications for practice and provide insights into targeted strategies to foster research capacity building and enhance research engagement among HPs, ultimately promoting evidence-based practice in healthcare. Based on our study the results, four major strategies can be considered:

1. Promoting the Value of Research in Clinical Care: Emphasising the attainment and intrinsic values of research can serve as a strong motivator for HPs to engage in research. Increasing awareness among HPs about the direct impact of research on their practice can foster a sense of purpose and intrinsic motivation [55]. This can be achieved through educational initiatives, highlighting success stories of research-driven improvements in patient care, and integrating research findings into clinical guidelines and practice. Healthcare organisations could demonstrate a commitment to valuing research by recognising and appreciating the contributions of HPs involved in research. This can be done through mechanisms such as dedicated research positions, research career pathways, and providing financial support for research activities [12]. Creating a supportive and encouraging environment for research within healthcare organisations can motivate HPs to engage in research and promote a supportive research culture.
2. Tailored Support for Early and Mid-Career HPs: Early career HPs face specific challenges and barriers to research engagement. Providing tailored support and resources to this group can help address their unique needs. This may include mentoring programs, research capacity-building workshops, and assistance with navigating the research process [56, 57]. Additionally, offering flexible research opportunities that allow for a work-life balance can encourage early and mid-career HPs to participate in research while maintaining their clinical responsibilities.
3. Addressing Gender Disparities: The study highlighted gender differences in the value attributed to research. To promote research engagement among all genders, it is essential to address gender disparities in research involvement [58]. This can be done by implementing gender-responsive strategies, such as acknowledging parenting responsibilities and ensuring equal access to research training, mentorship, and funding opportunities. Creating a supportive and inclusive research environment that values and recognises the contributions of all HPs, regardless of gender, is crucial.
4. Organisational Support and Culture: To foster research engagement among HPs, it is crucial to build a research-supportive organisational culture [59, 60]. This involves creating policies that prioritise research, providing necessary resources and infrastructure, including funding/grant opportunities, fostering collaboration and interdisciplinary research, and encouraging dissemination of research findings within and beyond the organisation. Additionally, offering professional development programs in research can help sustain research engagement among HPs at all career stages. To address the barriers related to lack of research skills and training support, it is essential for organisations to implement targeted research training programs [6]. These programs should be designed to cater to the specific needs of early and mid-career HPs and provide them with the necessary knowledge and skills to conduct research, and allocating protected research time can facilitate participation in such programs. Furthermore, establishing mentorship programs and promoting collaborative research opportunities can enhance research involvement among HPs. Pairing early and mid-career HPs with experienced researchers can provide guidance, support, and opportunities for hands-on research experience. Collaborative research projects involving multidisciplinary teams can foster a culture of research and promote cross-professional engagement.

### Strengths and limitations

The strengths of the study include the large data set and commensurate representativeness of the survey population of allied health, medical, nursing and midwifery HPs in early career, mid-career, and late career stages. The preponderance of female to male participants in our study reflects the heath workforce ratios. The use of the theoretical model EVC to develop survey questions to elicit responses provided in-depth understanding of the factors that influence the motivation of HPs to engage in research.

Nonetheless, generalisation and transferability of the study findings are limited by the fact that even though there was a high response rate from those who responded, and the required statistically powered sample size was achieved, only a 3.3% response rate of the total population was obtained. Therefore, the results should be applied with caution to other settings. Also, there were more respondents from one of the three study sites, and this could have led to sampling bias. Additionally, possibly only HPs who were interested in research chose to participate. However, there was a balance in terms of numbers of participants who had previously engaged with research/ training and those who had not. Furthermore, the online survey may not have captured the richness and depth of individual experiences. While the content analysis of the textual data may have been subjective, influenced by the researchers' interpretations. Finally, the survey may have been prone to social desirability bias. Nonetheless, triangulation of the close-ended survey results with the open-ended responses helped to reduce this type of bias. Additionally, the close-ended questions were kept unbiased through use of forced choice questions and neutral questions [61]. On the whole, integration of the findings from both phases has enhanced the depth of understanding by providing a more holistic view of the HPs' perceptions, allowing for increased validity of the study.

## Conclusion

This study has demonstrated that HPs' motivations and barriers to undertaking research vary depending on career stage, prior exposure to research training and involvement in research. Fostering research capacity building and enhancing research engagement among HPs requires targeted strategies that address the specific needs and barriers faced by different professional groups, career stages, and genders. In conclusion, promoting the value of research in clinical care, implementing targeted research training programs that address gender disparities and provide tailored support for early and mid-career HPs, building a research-supportive organisational culture that fosters mentorship programs and collaborative research opportunities are key strategies to foster research engagement among HPs and promote evidence-based practice in healthcare.

### Supplementary Information

**Supplementary Material 1.**

**Supplementary Material 2.**

## Data Availability

The data can be obtained from the corresponding author on reasonable request.

## Abbreviations

HPs: Health professionals
EVC: Expectancy-Value- Cost
AIHW: Australian Institute of Health and Welfare
HREC: Human Research Ethics Committee
CHHHS: Cairns Hospital and Hinterland Health Service
MHHS: Mackay Hospital and Health Service
THHS: Townsville Hospital and Health Service
HR: Human Resource
