# Supplementary material for: Exploring the motivation of health professionals to engage with research at various career stages
Source: BMC Health Serv Res. 2024 Mar 7;24:305. doi: 10.1186/s12913-024-10772-z (PMC10921689; doi:10.1186/s12913-024-10772-z)
Supplement: Supplementary file 1 — Supplementary Material 1. [file 12913_2024_10772_MOESM1_ESM.docx]

Additional file 1: Good Reporting of A Mixed Methods Study (GRAMMS) checklist

| **Guideline** | **Section and Line** |
| --- | --- |
| Describe the justification for using a mixed methods approach to the research question | Methods: Lines 142-151 |
| Describe the design in terms of the purpose, priority and sequence of methods | Methods: Lines 153-158 |
| Describe each method in terms of sampling, data collection and  analysis | Methods: Lines 169-226 |
| Describe where integration has occurred, how it has occurred and  who has participated in it | Methods: Lines 155-158  Results: Lines 317-360 |
| Describe any limitation of one method associated with the present of the other method | Discussion: Lines 507-522 |
| Describe any insights gained from mixing or integrating methods | Discussion: Lines 423-451 |

*Reference: O'Cathain A, Murphy E, Nicholl J. The quality of mixed methods studies in health services research. J Health Serv Res Policy. 2008;13: 92-98.*
