# Supplementary material for: Exploring the motivation of health professionals to engage with research at various career stages
Source: BMC Health Serv Res. 2024 Mar 7;24:305. doi: 10.1186/s12913-024-10772-z (PMC10921689; doi:10.1186/s12913-024-10772-z)
Supplement: Supplementary file 2 — Supplementary Material 2. [file 12913_2024_10772_MOESM2_ESM.docx]

**Additional file 2: Survey Questions**

| **Perceived importance of research** |
| --- |
| Research should be a high priority for a health professional |
| It is important to be kept informed about research findings |
| My routine work is influenced by evidence-based practice |
| Research findings are irrelevant to me as a practicing health professional |
| Research is important to improve patient care |
| Research is important for my recognition |
| Research is important for my self-satisfaction |
| **Attitude towards research** |
| I enjoy reading about clinical research studies in the literature |
| I would enjoy working on a research project related to my practice |
| I am confident in my ability to understand research and research terminologies related to my practice |
| I am confident in my ability to design research projects related to my practice |
| I am confident in my ability to evaluate research findings in terms of their application to my practice |
| Research is important in identifying and investigating problems in my practice |
| Research is important to clinical decision making |
| **Willingness to do research** |
| There are plenty of opportunities for me to take part in research |
| I would only participate in research if I had protected research time |
| I would only participate in research if I am paid |
| I would require supervision to do research |
| My daily work activities prevent me from doing research |
| I am prepared to make time to do research during working hours |
| I would like to undertake research in my professional area |
| **Motivator for research** |
| To improve the profession |
| Provide opportunity to learn more about disease management |
| Provide enhanced services to improve patient care |
| Provide financial reward |
| Interest in clinical research |
| Encouragement from a colleague/ mentor |
| Provide me with continuing professional development hours |
| Provide me with personal satisfaction |
| Availability of replacement for my research time |
| To support research |
| **Motivation to undertake research** |
| I am strongly motivated by the recognition I can earn from other people |
| I want other people to find out how good I really can be at my work |
| To me, success means doing better than other people |
| I am keenly aware of the promotion goals I have for myself |
| I am keenly aware of the income goals I have for myself |
| I enjoy tackling problems that are completely new to me |
| I enjoy trying to solve complex problems |
| The more difficult the problem, the more I enjoy trying to solve it |
| What matters most to me is enjoying what I do |
| It is important for me to be able to do what I enjoy most |
| **Perceived potential barriers to you undertaking/continuing research** |
| I don't believe research would benefit patient care |
| I don't believe research would benefit the organisation for which I work |
| Research is not valued in my work organisation |
| Lack of opportunity to research in my area of interest |
| Difficulty in finding a research supervisor/mentor |
| Achieving a work life balance |
| Family & carer commitments |
| Lack of funding for research |
| Lack of protected research time |
| Lack of access to research space/ equipment |
| Lack of research skills/ training/ support |
| Lack of access to individuals with appropriate ethics, research governance and grants expertise |
| Lack of access to library services for assistance with literature searching and document supply |
| Lower salary than a clinical career |
| Job insecurity relative to a clinical career |
| The thought of research makes me nervous |
|  |
